# Study of comparative performance of general-purpose LLM-based systems in predicting IVF outcomes

**DOI:** 10.1007/s10815-025-03793-y

**Published:** 2026-01-09

**Authors:** Can Dinç, Ömer Faruk Öz, Saltuk Buğra Arıkan, Selen Doğan, Murat Özekinci, Nasuh Utku Doğan, İnanç Mendilcioğlu

**Affiliations:** Department of Gynecology and Obstetrics, Akdeniz University, Antalya, Turkey

**Keywords:** In vitro fertilization, Artificial intelligence, Large language models, IVF protocol prediction, Oocyte count prediction, Clinical pregnancy prediction, Reproductive medicine

## Abstract

**Background and objective:**

Artificial intelligence (AI) has emerged as a promising tool for clinical decision support in reproductive medicine, yet the performance of general-purpose large language models (LLMs) in predicting in vitro fertilization (IVF) outcomes remains insufficiently characterized. This exploratory proof-of-concept study aimed to evaluate and compare the out-of-the-box performance of three widely accessible LLM-based systems (ChatGPT, DeepSeek, and Gemini) in forecasting key clinical and laboratory outcomes of IVF treatments.

**Methods:**

This retrospective single-center study used data from 1473 autologous IVF/ICSI cycles, each representing a unique patient. For each cycle, relevant clinical and laboratory variables were incorporated into a standardized anonymized patient-level vignette and submitted via the publicly available web interfaces of three LLMs (ChatGPT, DeepSeek, Gemini) without any fine-tuning or internal customization. The models were asked to predict stimulation protocol, ovulation trigger type, total and mature oocyte counts, usable embryo counts, and clinical pregnancy. Predictive performance was evaluated using accuracy and tolerance-based accuracy for categorical and count-based outcomes, mean absolute error for numerical predictions, and the area under the receiver operating characteristic (ROC) curve for clinical pregnancy.

**Results:**

Gemini achieved the highest accuracy in predicting stimulation protocols (51.26%) and embryo counts (68.22%), while DeepSeek demonstrated the lowest numerical error for oocyte count predictions. Clinical pregnancy prediction was the most challenging task; all models showed only moderate discrimination, with Gemini achieving the highest AUC (0.711), followed by ChatGPT (0.690) and DeepSeek (0.676). Overall, model performance varied considerably across tasks and remained below thresholds that would be considered sufficient for reliable stand-alone clinical use.

**Conclusions:**

In this exploratory proof-of-concept setting, general-purpose AI systems showed variable and overall suboptimal performance in predicting IVF outcomes from standardized clinical vignettes. Although certain models demonstrated relative strengths in specific tasks, none reached the reliability, consistency, or interpretability required for safe clinical implementation. These findings indicate that, in their current form, such models should not be used as clinical decision-support tools for IVF decision-making and that their use should remain restricted to carefully controlled research settings until they have been prospectively validated in multicenter cohorts and systematically compared with rigorously developed, task-specific prediction models. This study provides comparative insight into how these AI systems behave in IVF-related prediction tasks and underscores the need for cautious interpretation of AI-generated outputs.

**Supplementary Information:**

The online version contains supplementary material available at 10.1007/s10815-025-03793-y.

## Introduction

AI has increasingly become a fundamental element of modern medicine, offering new possibilities for enhancing clinical decision-making and improving patient care, including applications in diagnostics, risk stratification, treatment planning, and decision-support across multiple disciplines [1, 2]. Infertility affects approximately 8–12% of couples of reproductive age globally, driving the widespread use of assisted reproductive technologies (ART), particularly IVF [3, 4]. Although significant progress has been made in stimulation protocols, laboratory techniques, and embryo culture systems, IVF success rates remain limited, especially in cases involving diminished ovarian reserve or advanced maternal age [5, 6]. These limitations highlight the need for more individualized and data-driven approaches to treatment planning.

AI models offer the ability to analyze large, complex datasets and extract clinically meaningful patterns that may support decision-making at various stages of IVF, including protocol selection, oocyte yield prediction, embryo development, implantation potential, and pregnancy outcome estimation [7–9]. Most existing studies, however, have focused on conventional supervised machine learning or deep learning models trained directly on structured clinical or laboratory data. Many of these approaches are limited by single-center designs, heterogeneous methodologies, insufficient external validation, and a lack of systematic head-to-head comparisons between different AI strategies, which together restrict their real-world generalizability [9–12].

Previous studies have applied a range of machine learning and deep learning algorithms, including logistic regression, random forest, gradient boosting, support vector machines, artificial neural networks, and convolutional architectures, to predict outcomes such as ovarian response, embryo quality, clinical pregnancy, and live birth using structured clinical parameters, laboratory markers, and embryo or time-lapse imaging data [7–12]. Although several of these models have reported promising performance in specific settings, they are often not easily transferable between centers and are not routinely available as accessible, point-of-care tools in daily IVF practice. These limitations underline the need to explore how widely available AI systems might behave in realistic clinical scenarios.

ChatGPT, DeepSeek, and Gemini are general-purpose large language models designed to process and generate human-like text based on patterns learned from extensive training corpora. Although they are not specifically developed or fine-tuned for IVF outcome prediction, they are increasingly accessible to clinicians and patients and may be informally used to interpret clinical information or provide case-based suggestions. How such models perform when confronted with standardized patient-level IVF scenarios, and whether their outputs show any consistent alignment with real-world outcomes, has not yet been systematically evaluated.

To address this gap, this retrospective single-center study was designed as an exploratory, proof-of-concept benchmark of out-of-the-box performance of three widely accessible large language models, ChatGPT, DeepSeek, and Gemini, in the context of IVF care. Rather than developing new task-specific prediction algorithms or replacing conventional machine learning pipelines, we evaluated how these general-purpose models behave when provided with standardized, clinically realistic patient-level vignettes derived from routine IVF cycle data and asked to estimate key outcomes, including stimulation protocol category, ovulation trigger type, oocyte and embryo counts, and clinical pregnancy status. The objective of this study is to compare their predictive performance across these endpoints, to characterize their strengths and limitations, and to explore their behavior in IVF-related prediction tasks in order to inform the future development of rigorously designed, task-specific decision-support models, rather than to imply current clinical utility or equivalence to validated predictive models.

## Materials and methods

### Study design and data source

This retrospective single-center study analyzed autologous IVF/ICSI treatment cycles performed at a tertiary university-based assisted reproductive treatment center between October 2018 and April 2022. All consecutive cycles during this period were screened, and each included cycle corresponded to a unique patient; repeat cycles from the same patient were excluded to avoid within-patient clustering.

Cycles were eligible if they had complete data for predefined baseline, treatment, and outcome variables and a documented clinical pregnancy outcome. Donor cycles, fertility preservation cycles, and records with missing key data or internal inconsistencies were excluded. After applying these criteria, 1473 cycles were retained for analysis.

For each cycle, standardized data were collected on baseline clinical characteristics (female age, duration of marriage, duration of infertility, presence of chronic diseases, regular medication use, weight, height, body mass index [BMI, kg/m^2^], menstrual regularity, gravida, parity, number of abortions, history of curettage, history of ectopic pregnancy, and infertility etiology such as male factor, diminished ovarian reserve, endometriosis, tubal factor, or unexplained infertility); semen quality (total progressive motile sperm count, million); ovarian reserve and baseline hormones (antral follicle count; basal estradiol [E2, pg/mL]; follicle-stimulating hormone [FSH, IU/L]; luteinizing hormone [LH, IU/L]); treatment characteristics (ovarian stimulation protocol category: antagonist, progestin-primed ovarian stimulation, or agonist; ovulation trigger type: hCG, GnRH agonist, or dual trigger); and laboratory and outcome measures (total oocytes retrieved, number of mature [M2] oocytes, number of usable embryos, embryo transfer performed yes/no, and clinical pregnancy defined as the presence of an intrauterine gestational sac with fetal heartbeat).

**Fig. 1:**
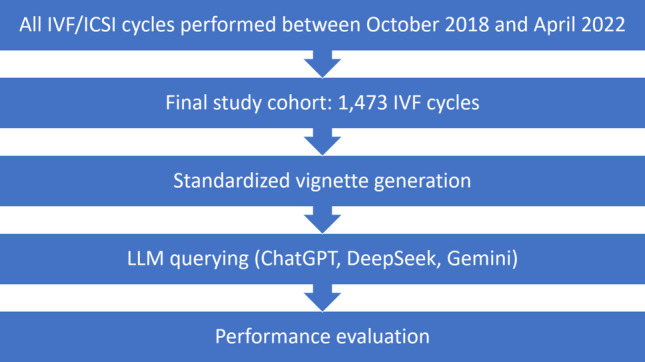
Study flow diagram of cohort selection, vignette generation, LLM querying (ChatGPT, DeepSeek, Gemini), and performance evaluation

A schematic flow diagram of cohort selection, vignette construction, LLM querying, and outcome evaluation is presented in Fig. 1.

### AI model input and task description

Three general-purpose large language models, ChatGPT (paid version), DeepSeek (free version), and Gemini (free version), were evaluated in this study based on their ability to process clinical information presented in natural language. All models were accessed via publicly available web interfaces without fine-tuning, additional training, or internal customization.

For each IVF cycle, the relevant anonymized clinical and laboratory variables were converted into a standardized, structured narrative vignette simulating a concise case summary. The same template was applied consistently for all three models. A typical input was formatted as follows:

“A 28-year-old woman, married for 2 years, with 1 year of infertility, no comorbidities, and no regular medication. Weight 52 kg, height 155 cm (BMI 21.6 kg/m^2^). Regular 28-day menstrual cycles. Gravida 0, para 0, abortion 0, ectopic pregnancy 0. Total progressive motile sperm count: 11.88 million. Antral follicle count: 12. Infertility etiology: diminished ovarian reserve and endometriosis. Previous treatments: 2 ovulation induction cycles and 1 embryo transfer. Basal E2: 46.0 pg/mL, basal LH: 2.9 IU/L, basal FSH: 5.7 IU/L. Based on this information, please predict: (a) the most appropriate stimulation protocol category, (b) the expected ovulation trigger type, (c) the expected total and mature oocyte counts, (d) the expected usable embryo count, and (e) the likelihood of achieving a clinical pregnancy in percentage.”

Each model was thus presented with one vignette per cycle and asked to generate predictions for the following predefined target outcomes:

1. Ovarian stimulation protocol category: antagonist, progestin-primed ovarian stimulation (PPOS), or agonist protocol.
2. Ovulation trigger type: human chorionic gonadotropin (hCG) alone, gonadotropin-releasing hormone (GnRH) agonist alone (agonist trigger), or combined hCG + GnRH agonist (dual trigger).
3. Total oocyte count (numeric).
4. Mature (M2) oocyte count (numeric).
5. Usable embryo count (numeric).
6. Clinical pregnancy: predicted probability (%) and corresponding binary outcome (yes/no).

The outputs provided by the models were extracted and numerically encoded according to a predefined coding scheme and then compared with actual clinical records for each outcome. This design was chosen deliberately to emulate a realistic decision-support setting in which LLMs interpret patient-level information in natural language, rather than operating as conventional regression or classification models directly trained on tabular data.

### Data preparation

All patient identifiers were removed prior to analysis. Only cycles with complete data for the predefined clinical and laboratory variables were included, resulting in a final sample of 1473 IVF cycles. For each cycle, the selected variables were mapped into the standardized vignette template described above. No additional external data sources or model-specific feature engineering were used. The same set of vignettes was submitted to each model.

### Outcome measures

Primary outcomes included prediction accuracy for stimulation protocol and trigger type, mean absolute error (MAE) and mean squared error (MSE) for oocyte and embryo counts, and classification accuracy for clinical pregnancy prediction. Tolerance margins of ± 3 oocytes for oocyte counts and ± 1 embryo for embryo counts were applied as exploratory, error-tolerant metrics to account for expected biological and laboratory variability and to facilitate comparative assessment of model behavior; these thresholds were not intended to imply clinical equivalence, and all primary interpretations are based on continuous performance metrics.

### Statistical analysis

All model predictions were systematically compared with the corresponding real-world IVF outcomes in our dataset. Model performance for continuous outcomes was assessed using mean absolute error (MAE), mean squared error (MSE), and R-squared (*R*^2^) values. For categorical outcomes (stimulation protocol, trigger type, and clinical pregnancy) and for tolerance-based evaluations of count outcomes, classification performance was evaluated using accuracy within predefined tolerance ranges where applicable. Chi-square tests were used to compare overall accuracy differences among models, and pairwise differences in model-based classifications were examined using McNemar tests to evaluate the statistical significance of discordant predictions. Receiver operating characteristic (ROC) curve analysis was performed for clinical pregnancy prediction, and area under the curve (AUC) values were reported.

All statistical analyses were performed using IBM SPSS Statistics version 26.0 (IBM Corp., Armonk, NY, USA), and a two-sided *p*-value of less than 0.05 was considered statistically significant. After completion of the quantitative analyses, the overall pattern of model performance and representative discrepancies between predictions and observed outcomes were jointly reviewed by senior reproductive medicine specialists among the co-authors to ensure that the interpretations were clinically plausible; however, no additional human adjudication was used to modify model outputs.

## Results

### Baseline characteristics

This study included 1473 IVF cycles, each representing a unique patient. Table 1 summarizes the baseline demographic, clinical, and laboratory characteristics of the cohort. The mean patient age was 33.18 ± 5.61 years, and the average BMI was 25.31 ± 5.32 kg/m^2^. The mean duration of infertility was 3.80 ± 3.33 years. The mean antral follicle count was 11.43 ± 9.86. Basal hormone levels were as follows: FSH 8.20 ± 5.59 mIU/mL, LH 5.49 ± 4.49 mIU/mL, and estradiol (E2) 63.57 ± 64.70 pg/mL. The mean number of mature (M2) oocytes retrieved was 7.12 ± 6.15, and the average number of embryos obtained was 1.33 ± 1.27.

**Table 1:** Baseline demographic, clinical, and laboratory characteristics (mean ± SD)

Variable	Mean ± SD
Age (years)	33.18 ± 5.61
BMI (kg/m^2^)	25.31 ± 5.32
Gravida (*n*)	0.57 ± 1.84
Parity (*n*)	0.15 ± 0.47
Abortus (*n*)	0.25 ± 0.63
Infertility duration (years)	3.80 ± 3.33
Total progressive sperm count (million)	35.76 ± 52.48
Antral follicle count (*n*)	11.43 ± 9.86
Basal E2 (pg/mL)	63.57 ± 64.70
Basal LH (mIU/mL)	5.49 ± 4.49
Basal FSH (mIU/mL)	8.20 ± 5.59
M2 oocyte count	7.12 ± 6.15
Embryo count	1.33 ± 1.27

Categorical distributions of stimulation protocols, ovulation trigger methods, and clinical pregnancy outcomes are summarized in Table 2. Approximately half of the patients underwent an antagonist protocol (50.2%), while the remaining received progestin-primed ovarian stimulation (PPOS) (49.8%). The dual trigger approach was used in the majority of cases (89.5%), followed by agonist trigger (8.2%) and hCG alone (2.3%). The overall clinical pregnancy rate in the study population was 21.8%.

**Table 2:** Categorical distributions (*n*, %)

Stimulation protocol	Trigger type	Clinical pregnancy
Antagonist: 739 (50.2%)	Dual trigger: 1318 (89.5%)	Positive: 321 (21.8%)
PPOS: 734 (49.8%)	Agonist: 121 (8.2%)	Negative: 1152 (78.2%)
	hCG: 34 (2.3%)	

Dual trigger = combined GnRH agonist + hCG; agonist trigger = GnRH agonist alone; hCG trigger = hCG alone

### Protocol prediction

The predictive performance of the three AI models (ChatGPT, DeepSeek, and Gemini) in identifying the appropriate ovarian stimulation protocol was evaluated using data from 1473 IVF cycles. Accuracy rates for each model were calculated and compared. A chi-square test of independence was performed to determine whether the differences in predictive accuracy among the models were statistically significant, as detailed in Table 3.

**Table 3:** Predictive accuracy of AI models for ovarian stimulation protocol selection (*n* = 1473)

Model	Correct prediction	Incorrect prediction	Accuracy (%)
ChatGPT	529	944	35.91
DeepSeek	588	885	39.92
Gemini	755	718	51.26

Among the evaluated models, Gemini achieved the highest accuracy in protocol prediction, correctly identifying the stimulation protocol in 51.26% of cases. This performance exceeded that of DeepSeek (39.92%) and ChatGPT (35.91%). The chi-square analysis confirmed that the differences in accuracy across models were statistically significant (*χ*^2^ = 76.41, df = 2, *p* < 0.001), indicating that the models varied meaningfully in their ability to predict stimulation protocols.

### Trigger type prediction

The models’ ability to predict the ovulation trigger type was also assessed using the same dataset. ChatGPT showed the highest accuracy at 39.31%, followed by DeepSeek at 26.61% and Gemini at 9.57%, as shown in Table 4. The chi-square test of independence revealed statistically significant differences among the models (*χ*^2^ = 348.12, df = 2, *p* < 0.001), suggesting that each model exhibited a distinct predictive pattern in determining the appropriate trigger method.

**Table 4:** Performance of AI models for predicting ovulation trigger type (dual trigger, GnRH agonist, or hCG) in 1473 IVF cycles

Model	Correct prediction	Incorrect prediction	Accuracy (%)
ChatGPT	579	894	39.31
DeepSeek	392	1081	26.61
Gemini	141	1332	9.57

Overall chi-square test: *χ*^2^ = 348.12, df = 2, *p* < 0.001

### Total oocyte count prediction

The models were further assessed for their ability to predict total oocyte count across 1473 IVF cycles. DeepSeek demonstrated the best numerical accuracy, with the lowest MAE = 5.25 and MSE = 1377.69, as presented in Table 5. However, all models yielded low *R*^2^ values (0.01–0.02), indicating limited capacity to explain variance in oocyte yield. Gemini and ChatGPT had comparable *R*^2^ scores, but slightly lower or higher absolute errors, respectively.

**Table 5:** Prediction performance of AI models for total oocyte count (*n* = 1473)

Model	MAE	MSE	*R*^2^ score	Correct predictions (± 3)	Accuracy (%)
ChatGPT	5.29	1367.32	0.02	673	45.69%
DeepSeek	5.25	1377.69	0.01	643	43.65%
Gemini	5.44	1380.92	0.01	687	46.66%

Additionally, prediction accuracy was evaluated using a ± 3-oocyte margin to classify outcomes as correct or incorrect. Within this threshold, Gemini achieved the highest classification accuracy (46.66%), followed by ChatGPT (45.69%) and DeepSeek (43.65%). Despite these differences, the chi-square test indicated that the variation in classification accuracy was not statistically significant (*χ*^2^ = 2.77, df = 4, *p* = 0.5972).

### M2 oocyte count prediction

The ability of the AI models to predict the number of mature (M2) oocytes’ count was evaluated using both regression metrics and classification accuracy within a ± 3 oocyte margin. As summarized in Table 6, DeepSeek yielded the lowest mean MAE = 3.52 and MSE = 26.45, indicating greater numerical precision. However, Gemini achieved the highest classification accuracy at 56.14%, slightly outperforming DeepSeek (54.23%) and ChatGPT (48.46%). Despite these differences, *R*^2^ scores were consistently low across all models, suggesting limited ability to account for inter-patient variability in M2 oocyte yield.

**Table 6:** M2 oocyte count prediction metrics and binary classification results (*n* = 1473)

Model	MAE	MSE	*R*^2^ score	Correct predictions (± 3)	Accuracy (%)
ChatGPT	3.62	25.50	0.32	714	48.46
DeepSeek	3.52	26.45	0.30	799	54.23
Gemini	3.66	29.59	0.22	827	56.14

### Embryo count prediction

Embryo count prediction performance was evaluated across the three models using 1473 IVF cycles. As presented in Table 7, Gemini demonstrated the best overall performance, with a mean absolute error of 2.46, a mean squared error of 10.83, and the highest classification accuracy of 68.22% within a margin of plus or minus one embryo. Although all models yielded negative *R*^2^ values, indicating limited explanatory power, Gemini had the least negative value at − 5.69, reflecting relatively better alignment with the actual embryo counts. These findings highlight the inherent complexity of embryo development and suggest that incorporating additional predictive variables may improve model performance. For this outcome, “correct” classification refers to predictions within ± 1 embryo of the observed usable embryo count, applied as an exploratory tolerance to compare models rather than as a definition of clinical interchangeability.

**Table 7:** Embryo count prediction metrics and binary classification results (*n* = 1473)

Model	MAE	MSE	*R*^2^ score	Correct predictions (± 1)	Accuracy (%)
ChatGPT	3.56	20.54	− 11.68	655	44.45
DeepSeek	2.58	12.29	− 6.58	910	61.76
Gemini	2.46	10.83	− 5.69	1005	68.22

### Clinical pregnancy prediction

The predictive performance of the three AI models in estimating clinical pregnancy was evaluated using normalized probability scores ranging from 0 to 1, compared against actual binary outcomes for pregnancy (0 = no pregnancy, 1 = pregnancy). As shown in Table 8, Gemini provided the best overall performance. While ChatGPT achieved the lowest mean absolute error at 0.305, Gemini produced the lowest mean squared error at 0.166 and was the only model to yield a positive *R*^2^ value (0.027). These results indicate that although ChatGPT minimized average error more effectively, Gemini’s predictions were more consistent and explained a greater proportion of the outcome variance. DeepSeek showed the weakest performance across all metrics. Taken together, these findings suggest that, although Gemini showed the best overall performance for clinical pregnancy prediction, the absolute predictive power of all three models remains insufficient for standalone clinical decision-making.

**Table 8:** Normalized regression metrics for clinical pregnancy prediction

Model	MAE	MSE	*R*^2^ score	Accuracy (%)
ChatGPT	0.305	0.212	− 0.244	72.44
DeepSeek	0.380	0.208	− 0.223	67.96
Gemini	0.319	0.166	0.027	78.28

### ROC analysis

ROC curve analysis was conducted to evaluate the discriminatory performance of the models. The area under the curve (AUC) values were 0.711 for Gemini, 0.690 for ChatGPT, and 0.676 for DeepSeek. While all three models showed moderate classification ability, Gemini achieved the highest AUC, supporting its relative superiority in distinguishing between pregnant and non-pregnant cases.

### Pairwise model comparison

To further compare the predictive behaviors of the AI models across different IVF-related tasks, pairwise McNemar tests were conducted for six outcome categories: protocol selection, trigger type, total oocyte count, M2 oocyte count, embryo count, and clinical pregnancy prediction. These analyses showed that Gemini significantly outperformed both ChatGPT and DeepSeek for protocol selection and embryo count prediction. Trigger type prediction exhibited the greatest divergence among models, with highly significant *p*-values for all pairwise comparisons. In contrast, most pairwise tests related to total and M2 oocyte count predictions did not reach statistical significance, suggesting more similar model behavior in those domains. For clinical pregnancy prediction, Gemini significantly outperformed DeepSeek, whereas its performance relative to ChatGPT did not differ significantly.

## Discussion

This retrospective study compared the predictive performance of three widely accessible AI models (ChatGPT, DeepSeek, and Gemini) in forecasting key clinical and laboratory outcomes in IVF using standardized patient-level vignettes derived from real-world cycle data. The results revealed that predictive performance varied notably across different outcomes, highlighting model-specific strengths and weaknesses and reinforcing the exploratory, hypothesis-generating nature of this evaluation.

Gemini showed the highest accuracy in predicting stimulation protocols, reaching 51.26%. Although statistically superior to the other models, this level remains insufficient for dependable clinical application, underscoring the need for further model refinement, comparison with conventional machine learning approaches, and external validation using independent datasets [12]. In ovulation trigger type prediction (hCG alone, GnRH agonist alone, or dual trigger with both), ChatGPT performed best with an accuracy of 39.31%, but this level remains insufficient to guide individualized trigger decisions, which depend on multifactorial clinical and physiological considerations.

For total oocyte count prediction, DeepSeek demonstrated the greatest numerical precision, as indicated by the lowest mean absolute and squared errors, whereas Gemini achieved slightly higher classification accuracy within the predefined tolerance range. Across all models, however, low *R*^2^ scores suggested limited capacity to capture the biological variability of ovarian response, reflecting the inherent complexity of controlled ovarian stimulation. Compared with previous work using task-specific models trained on structured cycle data, our results were more modest [13]. For example, Houri et al. developed an XGBoost-based model to predict high oocyte maturation rate (≥ 80%) in flexible GnRH antagonist cycles and reported an accuracy of approximately 75% and an AUC of 0.78, illustrating that dedicated, protocol-specific machine learning models can achieve substantially better performance than the out-of-the-box general-purpose LLMs evaluated in our study [13].

In mature (M2) oocyte count estimation, Gemini again yielded the highest classification accuracy, while DeepSeek provided more precise numerical outputs. Nonetheless, persistently low *R*^2^ values indicated that none of the models adequately accounted for interpatient variability in follicular development, further supporting the interpretation that their use in this context should remain exploratory rather than definitive.

Embryo count prediction was best handled by Gemini, which achieved 68.22% classification accuracy and the lowest mean errors. Although *R*^2^ scores remained negative across all models, Gemini exhibited the least deviation from observed values. While these findings indicate some alignment between predicted and observed embryo counts within predefined ranges, the overall performance remains insufficient to justify any clinical reliance on these models, even in a complementary role [14].

Clinical pregnancy prediction proved to be the most challenging task. Gemini was the only model to produce a positive *R*^2^ value and demonstrated the highest area under the ROC curve (AUC 0.711). ChatGPT and DeepSeek followed with slightly lower AUC values (0.690 and 0.676, respectively). Despite these findings, predictive performance remained limited, likely influenced by the complex, multifactorial nature of implantation and the imbalanced distribution of pregnancy outcomes in the dataset. While other studies have reported higher prediction accuracy using tailored machine learning models and different endpoints, our results were comparatively conservative [15, 16]. Several previous studies using dedicated machine learning or deep learning models, often based on carefully selected clinical features or embryo imaging, have reported higher discrimination for pregnancy or live birth prediction than observed in our analysis [15, 16]. For example, Goyal et al. reported a highest AUC of 0.846 and F1-score of 76.49% for live-birth prediction using a random forest classifier on a large HFEA dataset, which is higher than the AUC values achieved by the out-of-the-box LLMs in our study and underscores the advantage of task-specific models trained on large structured datasets [16].

In contrast, the general-purpose LLMs evaluated in this study were applied in an out-of-the-box manner without task-specific training on our local dataset, which likely contributes to their more modest performance and supports interpreting our findings as exploratory rather than as evidence that these models are ready for routine clinical deployment [17]. Moreover, although live birth is the ideal endpoint in IVF research, clinical pregnancy was used here due to incomplete follow-up data.

Pairwise comparisons using McNemar’s test revealed significant differences in predictive behavior between ChatGPT and DeepSeek, and between DeepSeek and Gemini. However, no statistically significant difference was observed between ChatGPT and Gemini for clinical pregnancy prediction. These differences likely reflect the fact that ChatGPT, DeepSeek, and Gemini are built on distinct architectures and training corpora and use different proprietary optimization and sampling strategies, so that “LLM performance” is inherently model-specific and findings from one system cannot be assumed to generalize directly to others.

As the application of AI in embryo selection and IVF optimization continues to evolve rapidly, and as general-purpose LLMs themselves are relatively new and frequently updated tools with limited systematic, clinically oriented comparisons across multiple IVF-related outcomes, our findings emphasize the variability and context dependency of model performance [18, 19]. This variability highlights the necessity for rigorous, standardized comparative evaluations before any AI tool, including general-purpose LLMs, can be responsibly integrated into routine clinical workflows, not only based on numerical performance metrics but also with respect to transparency, robustness, and clinical interpretability of their outputs. Beyond algorithmic innovation, successful integration demands evidence-based oversight, external validation, clear reporting of model inputs and prompts, and cautious, context-aware interpretation of LLM outputs by clinicians.

Despite technological advances, inconsistency in accuracy and limited transparency regarding model architecture and training data remain key barriers to clinical implementation [20]. Reliable use of AI in IVF will require standardized evaluation frameworks, multicenter validation, and clearer insight into how predictions are generated. Overall, this study should therefore be understood as an exploratory, proof-of-concept evaluation that maps the current capabilities and limitations of widely accessible, out-of-the-box general-purpose LLMs in the IVF context and is intended to motivate the development and rigorous validation of task-specific prediction models, rather than to recommend immediate clinical use of any of the systems evaluated here.

### Limitations

This study has several limitations. First, it is based on a single-center, retrospective cohort from one IVF clinic, which may limit the generalizability of the findings to broader populations and healthcare systems. Second, the distribution of outcomes was imbalanced, with clinical pregnancy substantially less frequent than non-pregnancy, which may have contributed to the modest discrimination and calibration of all evaluated models, particularly for pregnancy prediction, and we did not apply any explicit statistical correction for this class imbalance. Third, the large language models evaluated here are general-purpose systems that were not specifically designed or fine-tuned for structured predictive modeling of IVF outcomes; in our design, they were used as off-the-shelf, case-level reasoning tools operating on standardized narrative vignettes without any IVF-specific retraining on local data, which represents an important conceptual and methodological constraint. In addition, the proprietary and evolving nature of these models and the lack of full transparency regarding their training corpora mean that they effectively operate as black boxes whose predictions may partly reflect prior exposure to published IVF knowledge, and exact replication of our results with future model versions may not be possible.

Another key limitation is that we did not develop or include conventional multivariable or machine-learning/deep-learning models trained on the same dataset as task-specific comparators, which limits direct benchmarking and precludes any inference that the evaluated LLMs match or exceed the performance of optimized IVF prediction algorithms. Moreover, model performance was heterogeneous across endpoints: the systems aligned comparatively better with stimulation protocols and expected embryo counts, whereas performance was poorer for oocyte yield and especially for clinical pregnancy, likely reflecting both differences in the underlying signal-to-noise ratio of each task and the absence of several important prognostic factors (such as uterine characteristics, detailed embryo morphokinetics, and unmeasured lifestyle or genetic variables) from our vignettes. Finally, key endpoints such as live birth could not be fully assessed because of incomplete follow-up. Taken together, these limitations indicate that the present findings should be regarded as hypothesis-generating and cautionary, rather than as definitive evidence to support clinical implementation of out-of-the-box general-purpose LLMs in IVF decision-making.

## Conclusion

This exploratory study directly compared three widely accessible general-purpose large language models (ChatGPT, DeepSeek, and Gemini) in their ability to predict key IVF-related clinical and laboratory outcomes using standardized patient-level vignettes derived from real-world data. Performance varied substantially across tasks, and no model consistently achieved the accuracy, calibration, or reliability required for independent or routine clinical use.

In their current form, general-purpose LLMs are not suitable as clinical decision-support tools for IVF treatment planning, even in a complementary role. Their use should remain restricted to carefully controlled research settings, and they should not be relied upon for real-time clinical decision-making until they have been prospectively validated in multicenter cohorts and systematically benchmarked against rigorously developed, task-specific prediction models that include live birth and other clinically meaningful endpoints. These findings should be interpreted as hypothesis-generating and cautionary rather than as evidence supporting clinical implementation of current out-of-the-box LLMs in IVF.

Future work should focus on developing and validating task-specific prediction models based on large, representative IVF datasets that integrate structured clinical variables, embryology parameters, and, where appropriate, imaging data; systematically comparing such models with general-purpose LLM-based approaches; improving model transparency and interpretability; and establishing standardized evaluation frameworks to determine whether, and under which conditions, LLM-based tools can be safely and effectively incorporated into reproductive medicine.

## Supplementary Information

Below is the link to the electronic supplementary material.

ESM 1 Supplementary Material 1 (DOCX 14.2 KB)

## Data Availability

The data of this study is available at the Department of Gynecology and Obstetrics, Akdeniz University.
