# Supplementary material for: Study of comparative performance of general-purpose LLM-based systems in predicting IVF outcomes
Source: J Assist Reprod Genet. 2026 Jan 9;43(3):731–9. doi: 10.1007/s10815-025-03793-y (PMC12982683; doi:10.1007/s10815-025-03793-y)
Supplement: Supplementary file 1 — Supplementary Material 1 (DOCX 14.2 KB) [file 10815_2025_3793_MOESM1_ESM.docx]

**Supplementary Table S1. Overview of variables used for vignette construction and outcome assessment**

| **Category** | **Variable** | **Definition / Coding** | **Unit / Type** |
| --- | --- | --- | --- |
| Patient demographics | Age | Age at the start of IVF/ICSI cycle | Years (continuous) |
|  | Duration of marriage | Time from marriage to IVF cycle | Years (continuous) |
|  | Duration of infertility | Time attempting conception without success | Years (continuous) |
|  | Chronic diseases | Presence of chronic systemic disease (e.g. DM, HT) | Yes/No, categorical |
|  | Regular medication | Use of regular systemic medication | Yes/No, categorical |
|  | Weight | Measured at cycle start | kg |
|  | Height | Measured at cycle start | cm |
|  | BMI | Weight / height² | kg/m² (continuous) |
| Menstrual and obstetric history | Menstrual regularity | Cycle regularity (e.g. regular 25–35 days, irregular) | Categorical |
|  | Gravida | Number of prior pregnancies | Count |
|  | Parity | Number of prior births ≥20 weeks | Count |
|  | Abortion | Number of spontaneous or induced abortions | Count |
|  | Curettage history | History of uterine curettage | Yes/No |
|  | Ectopic pregnancy history | History of ectopic pregnancy | Yes/No |
| Male factor | Total progressive motile sperm count | Total progressively motile sperm in ejaculate used for IVF/ICSI | Million (continuous) |
| Ovarian reserve | Antral follicle count (AFC) | Total AFC at baseline ultrasound | Count |
| Infertility etiology | Infertility etiology | Primary cause(s): male factor, diminished ovarian reserve, endometriosis, tubal factor, unexplained, combined, other | Categorical (single or combined) |
| Treatment history | Previous controlled ovarian stimulation cycles | Number of prior COS cycles | Count |
|  | Previous embryo transfers | Number of prior embryo transfer procedures | Count |
| Basal hormones | Basal E2 | Estradiol on cycle day 2–3 | pg/mL (continuous) |
|  | Basal FSH | FSH on cycle day 2–3 | IU/L (continuous) |
|  | Basal LH | LH on cycle day 2–3 | IU/L (continuous) |
| Treatment protocol (observed) | Stimulation protocol | Actual protocol used: antagonist, progestin-primed ovarian stimulation (PPOS), long agonist, other agonist | Categorical |
|  | Ovulation trigger type | Actual trigger: hCG, GnRH agonist, dual trigger | Categorical |
| Laboratory outcomes | Total oocyte count | Total number of retrieved oocytes | Count |
|  | M2 oocyte count | Number of metaphase II oocytes | Count |
|  | Usable embryo count | Number of embryos deemed suitable for transfer or cryopreservation according to local criteria | Count |
| Clinical outcome | Clinical pregnancy | Presence of intrauterine gestational sac with fetal heartbeat on ultrasound | Binary (Yes/No) |
|  | Clinical pregnancy probability (LLM output) | Percentage likelihood estimated by each LLM based on vignette | Percent (model output) |
